# GPs’ familiarity with and use of cardiovascular clinical prediction rules: a UK survey study

**DOI:** 10.3399/bjgpopen20X101081

**Published:** 2020-10-07

**Authors:** Jong-Wook Ban, Rafael Perera, Richard Stevens

**Affiliations:** 1 Evidence-Based Health Care Programme, Centre for Evidence-Based Medicine, University of Oxford, Oxford, UK; 2 Nuffield Department of Primary Care Health Sciences, Medical Science Division, University of Oxford, Oxford, UK

**Keywords:** cardiovascular diseases, Clinical prediction rules, guidelines, general practice, surveys and questionnaires

## Abstract

**Background:**

Clinical prediction rules (CPRs) can help general practitioners (GPs) address challenges in cardiovascular disease. A survey published in 2014 evaluated GPs’ awareness and use of CPRs in the UK. However, many new CPRs have been published since and it is unknown which cardiovascular CPRs are currently recognised and used.

**Aim:**

To identify cardiovascular CPRs recognised and used by GPs, and to assess how GPs’ familiarity and use have changed over time.

**Design & setting:**

An online survey of GPs in the UK was undertaken.

**Method:**

Using comparable methods to the 2014 survey, GPs were recruited from a network of doctors in the UK. They were asked how familiar they were with cardiovascular CPRs, how frequently they used them, and why they used them. The results were compared with the 2014 survey.

**Results:**

Most of 401 GPs were familiar with QRISK scores, ABCD scores, CHADS scores, HAS-BLED score, Wells scores for deep vein thrombosis, and Wells scores for pulmonary embolism. The proportions of GPs using these CPRs were 96.3%, 65.1%, 97.3%, 93.0%, 92.5%, and 82.0%, respectively. GPs’ use increased by 31.2% for QRISK scores, by 13.5% for ABCD scores, by 54.6% for CHADS scores, by 33.2% for Wells scores for deep vein thrombosis, and by 43.6% for Wells scores for pulmonary embolism; and decreased by 45.9% for the Joint British Societies (JBS) risk calculator, by 38.7% for Framingham risk scores, and by 8.7% for New Zealand tables. GPs most commonly used cardiovascular CPRs to guide therapy and referral.

**Conclusion:**

The study found GPs’ familiarity and use of cardiovascular CPRs changed substantially. Integrating CPRs into guidelines and practice software might increase familiarity and use.

## How this fits in

Clinical prediction rules (CPRs) can help GPs address challenges in preventing and managing cardiovascular disease. The previous study from 2014 found cardiovascular CPRs used by the majority of GPs in the UK were Framingham risk scores, QRISK scores, and Wells scores for deep vein thrombosis. The present study showed GPs predominately used QRISK scores to assess cardiovascular disease risk; and used ABCD scores, CHADS scores, HAS-BLED score, Wells scores for deep vein thrombosis, and Wells scores for pulmonary embolism for stroke, and venous thromboembolism currently. Integrating high quality CPRs into national guidelines and GPs’ electronic health records (EHRs) might facilitate GPs’ familiarity with and use of them in practice.

## Introduction

Morbidity and mortality from cardiovascular disease are some of the leading sources of burden of disease in the UK,^1^ and pose many challenges to GPs. CPRs might help GPs address these challenges from cardiovascular disease by improving clinical outcomes, increasing efficiencies, and reducing costs.^2,3^

Although many cardiovascular CPRs exist,^4,5^ only a few have been broadly used by GPs.^6^ This may be owing to the following inefficiencies in cardiovascular CPR development. First, many authors do not justify why new CPRs are needed by examining existing CPRs,^7^ which often leads to the development of redundant CPRs. Second, most CPRs do not get evaluated in an independent external validation study even many years after they are developed.^8^ Third, CPRs evaluated in high quality impact studies are rare.^9^ These CPRs, without independently confirmed external validity and high quality evidence of impact, are unlikely to be recommended by guidelines or integrated in practice software, and therefore gain the trust of clinicians.

Electronic databases were searched and 23 studies were found that assessed the familiarity or use of various cardiovascular CPRs: 12 from European countries,^6,10–20^ six from the US,^21–26^ three from Australia and New Zealand,^27–29^ and two from other countries.^30,31^ These studies showed the familiarity with and use of cardiovascular CPRs varied widely according to specialties and geographic locations. For example, fewer GPs in Europe^20^ and primary care doctors in the US^26^ reported using CHA_2_DS_2_-VASc score compared with cardiologists. On the other hand, more GPs in Europe reported using HAS-BLED score than primary care doctors in the US.^20,26^

A UK study by Plüddemann *et al* ^6^ evaluated GPs’ awareness and use of CPRs for multiple clinical areas. The study included a survey of CPRs for cardiovascular disease risk, stroke, and venous thromboembolism that found the majority of GPs used Framingham risk scores, QRISK scores, and Wells rule for deep vein thrombosis.^6^ However, CPRs assessed in their study were primarily identified from guidelines developed before 2011 and many new CPRs have been published since.

Therefore, a survey was conducted to understand which cardiovascular CPRs are currently recognised and used by GPs in the UK. Furthermore, changes in the GPs’ familiarity with and use of cardiovascular CPRs were assessed by comparing the results of the current and 2014 survey by Plüddemann *et al*.^6^

## Method

The target participants were GPs who were practising medicine in the UK at the time of the survey. Doctors in training, GP registrars, and retired GPs were excluded. Using identical methods to the 2014 survey,^6^ GPs were recruited from doctors.net.uk (https://doctors.net.uk), which is a network of doctors in the UK with >238 000 members. Doctors.net.uk sent emails with an online link for the study information page to invite GP members who opted to receive information about research participation. The information page outlined the aim of the study, why they were invited, who the researchers were, how long it would take to complete, how data would be stored, and how to raise any concern. For participating, 1000 electronic surfing reward points were offered as an incentive, which is equivalent to 5 GBP. Participants confirmed they were aged ≥18 years, that they had read and understood the information page, and agreed to participate voluntarily by ticking a box before they could proceed to the questionnaire. Each GP could take part in the survey no more than once. To be comparable with the 2014 survey, the study aimed to recruit a convenience sample of 401 GPs stratified by geographic regions (or approximately 0.9% of GP members from each region). Doctors.net.uk closed the survey in a region once the target sample size for the region was reached.

The questionnaire consisted of three sections (see Supplementary Box S1). The first section included three questions for determining eligibility. The second section contained questions about the familiarity with and use of 19 cardiovascular CPRs. For the familiarity with CPRs, participants were asked to indicate whether: a) the CPR was integrated in electronic health record (EHR); b) they had heard of it; c) they had never heard of it; or d) they were not sure. For the use of CPRs, participants were asked to report whether they used the CPR: a) in most or all relevant cases; b) occasionally; c) rarely; or d) never. The order of CPRs presented to each survey participant was randomised. Participants were also asked to indicate for which specific reasons they used cardiovascular CPRs. The last section consisted of five questions about the demographics of participants. No personal information was collected. The questions and available answers were the same as the 2014 survey except for the additional option of 'the CPR was integrated in EHR' for the familiarity question.

Cardiovascular CPRs were selected that were likely to be recognised and used by GPs in the UK (Table 1). First, all CPRs for cardiovascular disease risk, stroke, and venous thromboembolism were included from the 2014 survey for comparability. When a CPR had been updated, the updated versions were chosen. Second, the American College of Cardiology (ACC) and American Heart Association (AHA) pooled cohort equation^32^ and New Zealand Primary Prevention Equations^33^ were included because major guidelines in the US^34^ and New Zealand^35^ currently recommend them instead of Framingham risk scores and New Zealand tables. Third, any additional cardiovascular CPRs recommended by major UK,^36–41^ European,^42–46^ and World Health Organization^47^ guidelines were included. GPs were also asked to name any other cardiovascular CPRs or CPRs in other clinical areas that they were using.

**Table 1.** Clinical prediction rules included in the current and 2014 survey

CPR included in the 2014 survey	CPR included in the current survey	Current guideline recommending CPR
Cardiovascular disease risk		
JBS2 risk charts or calculator^67^	JBS3 risk calculator^68^	JBS’ consensus recommendations for the prevention of cardiovascular disease (JBS3)^68^
QRISK or QRISK2^69,70^	QRISK2 or QRISK3^70,71^	NICE clinical guideline CG181: Cardiovascular disease: risk assessment and reduction, including lipid modification^36^
ASSIGN score^72^	ASSIGN score^72^	SIGN 149: Risk estimation and the prevention of cardiovascular disease^37^
UKPDS risk engine^73^	UKPDS risk engine^73^	—
SCORE risk charts^74^	SCORE risk charts^74^	2016 European guidelines on cardiovascular disease prevention in clinical practice^42^
PROCAM score^75^	PROCAM score^75^	—
Framingham risk scores^76^	Framingham risk scores^76^	—
—	ACC/AHA pooled cohort equation^32^	2019 ACC/AHA guideline on the primary prevention of cardiovascular disease^34^
New Zealand tables^77^	New Zealand tables^77^	—
—	New Zealand primary prevention equations^33^	New Zealand Ministry of Health: Cardiovascular disease risk assessment and management for primary care 2018^35^
—	WHO/ISH risk prediction charts^78^	WHO: Prevention of cardiovascular disease^47^
Stroke and venous thromboembolism^a^		
ABCD or ABCD^2^ ^79,80^	ABCD^2^, ABCD^3^, or ABCD^3^-I score^80,81^	NICE clinical guideline CG68: Stroke and transient ischaemic attack in over 16 seconds: diagnosis and initial management^82^
California score^83^	California score^83^	—
CHADS or CHADS_2_^84^	CHADS_2_ or CHA_2_DS_2_-VASc score^84,85^	NICE clinical guideline CG180: Atrial fibrillation: management;^38^ SIGN 129: Antithrombotics: indications and management;^39^ and 2016 ESC guidelines for the management of atrial fibrillation developed in collaboration with EACTS^43^
—	HAS-BLED score^86^	NICE clinical guideline CG180: Atrial fibrillation: management^38^
Wells scores for deep vein thrombosis^87^	Wells scores for deep vein thrombosis^87^	NICE clinical guideline CG144: Venous thromboembolic diseases: diagnosis, management and thrombophilia testing;^40^ and SIGN 122: Prevention and management of venous thromboembolism^41^
Wells score for pulmonary embolism^88^	Wells scores for pulmonary embolism^88^	NICE clinical guideline CG144: Venous thromboembolic diseases: diagnosis, management and thrombophilia testing;^40^ and SIGN 122: Prevention and management of venous thromboembolism^41^
—	Geneva or revised Geneva score^89,90^	2014 ESC guidelines on the diagnosis and management of acute pulmonary embolism;^44^ and SIGN 122: Prevention and management of venous thromboembolism^41^
—	Pulmonary Embolism Severity Index (PESI) or simplified Pulmonary Embolism Severity Index (sPESI)^91,92^	2014 ESC guidelines on the diagnosis and management of acute pulmonary embolism^45^

^a^These CPRs were included under the ’General Medical’ category in the 2014 survey. ACC/AHA = American College of Cardiology/Amercian Heart Association. CPR = clinical prediction rule. ESC = European Society of Cardiology. ISH = International Society of Hypertension. JBS = Joint British Societies. NICE = National Institute for Health and Care Excellence. SIGN = Scottish Intercollegiate Guidelines Network. WHO = World Health Organization.

The characteristics of participants were described using medians and interquartile ranges for continuous variables, and numbers and proportions for categorical variables. Two-sample test of proportions were used to assess the null hypotheses that proportions of GPs unfamiliar with CPRs and proportions of GPs using CPRs are the same between the current and 2014 survey. Because conducting multiple significance tests (11 comparisons for GPs’ familiarity with CPRs and 11 comparisons for GPs’ use of CPRs) can increase the risk of type I error, the robustness of the results were examined by adjusting *P* values and confidence intervals (CIs) using the Bonferroni method.^48^ The reasons for using CPRs were presented with numbers and proportions. Stata (version 14) was used for all analyses.

## Results

The survey commenced on 18 June 2019 and concluded on 1 July 2019 when the target sample size of 401 was reached. Characteristics of GPs who participated and their practices were similar between the current and 2014 survey (Table 2). Compared with the 2014 survey, the proportions of GPs unfamiliar with QRISK scores, ABCD scores, CHADS scores, Wells scores for deep vein thrombosis, and Wells scores for pulmonary embolism decreased (Table 3).

**Table 2.** Characteristics of GPs and their practices, *N* = 401

Characteristic	2014 survey, *n* (%)^a^	2019 survey, *n* (%)^a^
Sex^b^		
Male	243 (60.6)	245 (61.1)
Female	158 (39.4)	153 (38.2)
Other	—	3 (0.7)
Median year qualified (IQR)	1995 (1986–2000)	1998 (1991–2004)
Type of GP		
GP partner or principal	267 (66.6)	222 (55.4)
Salaried GP	95 (23.7)	117 (29.2)
Locum GP	32 (8.0)	54 (13.5)
Sessional GP	4 (1.0)	8 (2.0)
Retainer GP	3 (0.7)	0 (0.0)
Academic role^c^		
Research only	—	7 (1.7)
Teaching only	—	197 (49.1)
Both research and teaching	—	38 (9.5)
Neither	—	159 (39.7)
Median practice size^d^ (IQR)	6 GPs (4 to 7)	6 GPs (4 to 8)
Practice type		
Urban area	150 (37.4)	161 (40.1)
Suburban area	119 (29.7)	105 (26.2)
Semi-rural area	87 (21.7)	89 (22.2)
Rural area	45 (11.2)	46 (11.5)
Region^e^		
England	323 (80.5)	335 (83.5)
East of England	35 (8.7)	34 (8.5)
London	48 (12.0)	56 (14.0)
East Midlands	25 (6.2)	26 (6.5)
West Midlands	33 (8.2)	36 (9.0)
North East	17 (4.2)	16 (4.0)
Yorkshire & Humber	33 (8.2)	33 (8.2)
North West	43 (10.7)	43 (10.7)
South East	51 (12.7)	54 (13.5)
South West	38 (9.5)	37 (9.2)
Scotland	48 (12.0)	38 (9.5)
Wales	18 (4.5)	17 (4.2)
Northern Ireland	12 (3.0)	11 (2.7)

^a^Unless stated otherwise. ^b^'Other' option was not available in the 2014 survey. ^c^Academic role of GP was not included in the 2014 survey. ^d^16 of 401 GPs did not know practice size in the current survey. ^e^England was further stratified by the strategic health authority regions of the NHS. IQR = interquartile range.

**Table 3.** GPs unfamiliar with clinical prediction rules (CPRs): GPs who had never heard of or who were not sure whether they heard of respective CPRs, *N* = 401

CPR	2014 survey, *n* (%)	2019 survey, *n* (%)	Change in proportion of GPs unfamiliar to CPR
'Never heard of'	'Not sure'	Total	'Never heard of'	'Not sure'	Total	%	95% CI	*P* value	99.5% CI^a^	*P* value^a^
**Cardiovascular disease**											
JBS3 risk calculator^b^	19 (4.7)	8 (2.0)	27 (6.7)	122 (30.4)	28 (7.0)	150 (37.4)	30.7	25.3 to 36.0	<0.001	23.0 to 38.3	<0.011
QRISK2 or QRISK3^c^	64 (16.0)	24 (6.0)	88 (21.9)	4 (1.0)	0 (0.0)	4 (1.0)	–20.9	–25.1 to –16.8	<0.001	–26.9 to –15.0	<0.011
ASSIGN score	—	—	—	222 (55.4)	32 (8.0)	254 (63.3)	—	—	—	—	—
UKPDS risk engine	—	—	—	140 (34.9)	23 (5.7)	163 (40.6)	—	—	—	—	—
SCORE risk charts	265 (66.1)	67 (16.7)	332 (82.8)	273 (68.1)	37 (9.2)	310 (77.3)	–5.5	–11.0 to 0.0	0.052	–13.4 to 2.4	0.571
PROCAM score	328 (81.8)	50 (12.5)	378 (94.3)	337 (84.0)	36 (9.0)	373 (93.0)	–1.2	–4.6 to 2.1	0.469	–6.1 to 3.6	1.000
Framingham risk scores	4 (1.0)	2 (0.5)	6 (1.5)	18 (4.5)	4 (1.0)	22 (5.5)	4.0	1.5 to 6.5	0.002	0.4 to 7.6	0.023
ACC/AHA pooled cohort equation^d^	—	—	—	347 (86.5)	30 (7.5)	377 (94.0)	—	—	—	—	—
New Zealand tables	249 (62.1)	64 (16.0)	313 (78.1)	328 (81.8)	26 (6.5)	354 (88.3)	10.2	5.1 to 15.4	<0.001	2.9 to 17.6	<0.011
New Zealand primary prevention equations^d^	—	—	—	352 (87.8)	28 (7.0)	380 (94.8)	—	—	—	—	—
WHO/ISH risk prediction charts^d^	—	—	—	262 (65.3)	33 (8.2)	295 (73.6)	—	—	—	—	—
**Stroke and venous thromboembolism**											
ABCD^2^, ABCD^3^ or ABCD^3^-I score^e^	128 (31.9)	45 (11.2)	173 (43.1)	98 (24.4)	14 (3.5)	112 (27.9)	–15.2	–21.8 to –8.7	<0.001	–24.6 to –5.8	<0.011
California score	336 (83.8)	50 (12.5)	386 (96.3)	341 (85.0)	31 (7.7)	372 (92.8)	–3.5	–6.6 to –0.3	0.030	–8.0 to 1.0	0.329
CHADS_2_ or CHA_2_DS_2_-VASc score^f^	160 (39.9)	48 (12.0)	208 (51.9)	5 (1.2)	2 (0.5)	7 (1.7)	–50.1	–55.2 to –45.1	<0.001	–57.4 to –42.9	<0.011
HAS-BLED score^d^	—	—	—	12 (3.0)	4 (1.0)	16 (4.0)	—	—	—	—	—
Wells scores for deep vein thrombosis	109 (27.2)	30 (7.5)	139 (34.7)	12 (3.0)	5 (1.2)	17 (4.2)	–30.4	–35.5 to –25.4	<0.001	–37.7 to –23.2	<0.011
Wells scores for pulmonary embolism	169 (42.1)	59 (14.7)	228 (56.9)	31 (7.7)	21 (5.2)	52 (13.0)	–43.9	–49.7 to –38.0	<0.001	–52.3 to –35.5	<0.011
Geneva or revised Geneva score^d^	—	—	—	315 (78.6)	32 (8.0)	347 (86.5)	—	—	—	—	—
PESI or simplified PESI^d^	—	—	—	318 (79.3)	30 (7.5)	348 (86.8)	—	—	—	—	—

^a^Adjusted for conducting 11 significance tests using the Bonferroni method. ^b^JBS2 risk calculator or JBS2 risk charts' were included in the 2014 survey. ^c^'QRISK or QRISK2' were included in the 2014 survey. ^d^These CPRs were not included in the 2014 survey. ^e^'ABCD or ABCD^2^' were included in the 2014 survey. ^f^'CHADS or CHADS_2_' were included in the 2014 survey. ACC/AHA = American College of Cardiology/American Heart Association. CPR = clinical prediction rule. JBS = Joint British Societies. PESI = Pulmonary Embolism Severity Index. WHO/ISH = World Health Organization/International Society of Hypertension.

**Figure 1.**
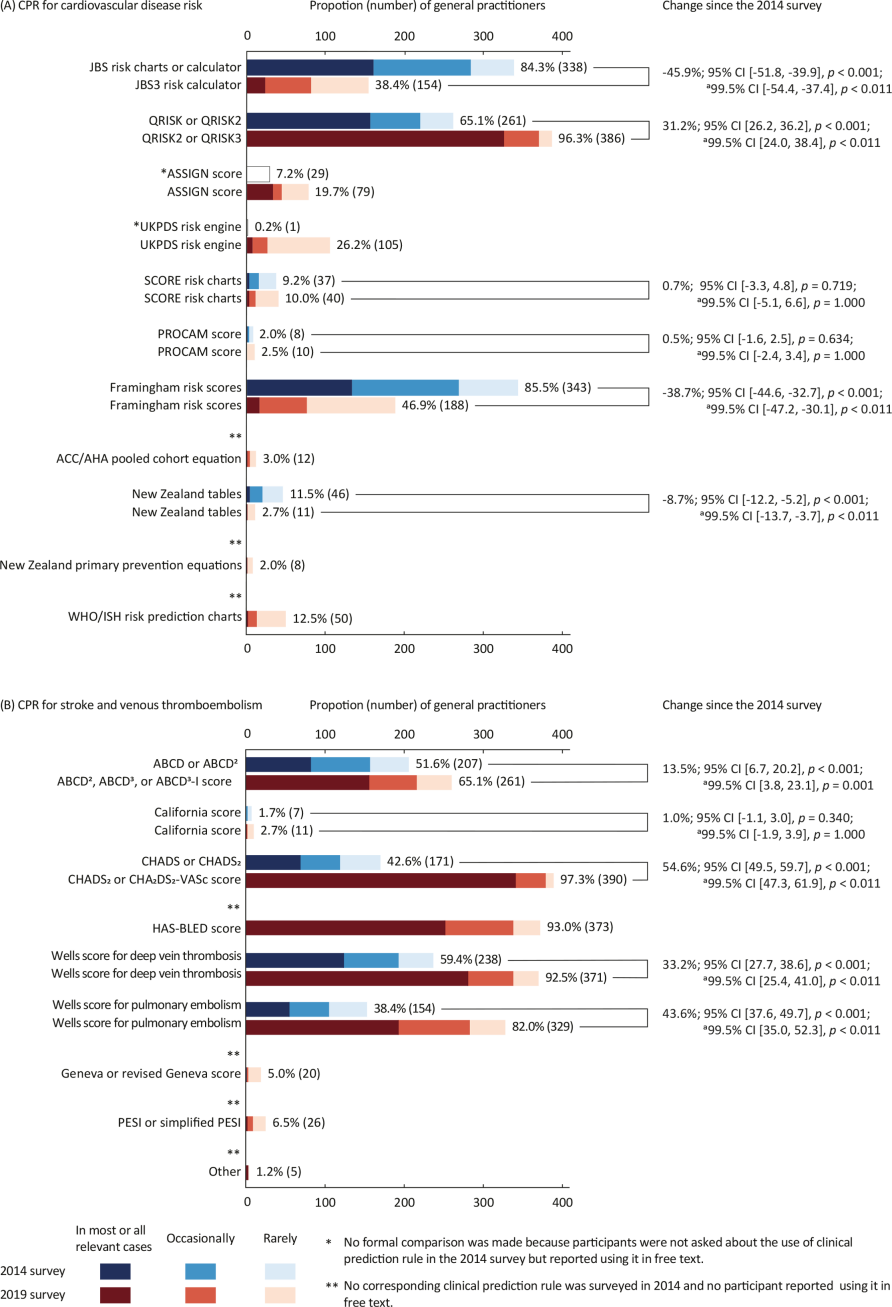
The proportion of GPs using clinical prediction rules for A) cardiovascular disease risk and B) venous thromboembolism in the 2014 and current survey. ^a^Confidence interval (CI) and *P* value adjusted using the Bonferroni method

The proportions of GPs using CPRs in the 2014 and current survey are presented in Figure 1. For cardiovascular disease risk CPRs, the proportion of GPs using JBS risk calculator, Framingham risk scores, and New Zealand tables decreased, whereas the proportion of GPs using QRISK scores increased (Figure 1A). Among CPRs for stroke and venous thromboembolism, the proportion of GPs using ABCD scores, California scores, CHADS scores, Wells scores for deep vein thrombosis, and Well scores for pulmonary embolism increased (Figure 1B). Five GPs named four other CPRs they were using for stroke and venous thromboembolism but which were not asked about in the survey. They were the Cincinnati Prehospital Stroke Scale (CPSS, also known as FAST)^49^ (*n* = 2), the National Institutes of Health (NIH) Stroke Scale^50^ (*n* = 1), the Pulmonary Embolism Rule-out Criteria (PERC) rule^51^ (*n* = 1), and QStroke score^52^ (*n* = 1).

The frequency of using cardiovascular CPRs is presented in Table 4. Many GPs reported using a CPR in most or all cases when it is integrated into their EHR software. Cardiovascular CPRs most often integrated into EHR software were QRISK scores, ABCD scores, CHADS scores, HAS-BLED score, Wells scores for deep vein thrombosis, and Wells scores for pulmonary embolism. The reported reasons for using CPRs are presented in Table 5.

**Table 4.** Frequency of using cardiovascular clinical prediction rules by all GPs who participated and GPs who reported that CPR was integrated in electronic health record

CPR	All GPs who participated in the survey	GPs who reported that 'CPR was integrated in electronic health record'
GPs, *n*	Use CPR, *n* (%)	Do not use CPR, *n* (%)	GPs, *n*	Use CPR, *n* (%)	Do not use CPR, *n* (%)
Most or all cases	Occasionally	Rarely	Most or all cases	Occasionally	Rarely
**Cardiovascular disease**										
JBS3 risk calculator	401	23 (5.7)	58 (14.5)	73 (18.2)	247 (61.6)	43	12 (27.9)	16 (37.2)	9 (20.9)	6 (14.0)
QRISK2 or QRISK3	401	325 (81.0)	44 (11.0)	17 (4.2)	15 (3.7)	280	257 (91.8)	21 (7.5)	1 (0.4)	1 (0.4)
ASSIGN score	401	33 (8.2)	11 (2.7)	35 (8.7)	322 (80.3)	24	21 (87.5)	2 (8.3)	1 (4.2)	0 (0.0)
UKPDS risk engine	401	7 (1.7)	19 (4.7)	79 (19.7)	296 (73.8)	14	3 (21.4)	3 (21.4)	4 (28.6)	4 (28.6)
SCORE risk charts	401	3 (0.7)	8 (2.0)	29 (7.2)	361 (90.0)	3	1 (33.3)	1 (33.3)	1 (33.3)	0 (0.0)
PROCAM score	401	0 (0.0)	0 (0.0)	10 (2.5)	391 (97.5)	2	0 (0.0)	0 (0.0)	0 (0.0)	2 (100)
Framingham risk scores	401	16 (4.0)	60 (15.0)	112 (27.9)	213 (53.1)	56	7 (12.5)	19 (33.9)	19 (33.9)	11 (19.6)
ACC/AHA pooled cohort equation	401	1 (0.2)	3 (0.7)	8 (2.0)	389 (97.0)	1	1 (100)	0 (0.0)	0 (0.0)	0 (0.0)
New Zealand tables	401	1 (0.2)	0 (0.0)	10 (2.5)	390 (97.3)	1	1 (100)	0 (0.0)	0 (0.0)	0 (0.0)
New Zealand primary prevention equation	401	1 (0.2)	0 (0.0)	7 (1.7)	393 (98.0)	1	1 (100)	0 (0.0)	0 (0.0)	0 (0.0)
WHO/ISH risk prediction charts	401	2 (0.5)	11 (2.7)	37 (9.2)	351 (87.5)	2	0 (0.0)	1 (50.0)	1 (50.0)	0 (0.0)
**Stroke and venous thromboembolism**										
ABCD^2^, ABCD^3^ or ABCD^3^-I score	401	156 (38.9)	61 (15.2)	44 (11.0)	140 (34.9)	107	81 (75.7)	21 (19.6)	3 (2.8)	2 (1.9)
California score	401	1 (0.2)	2 (0.5)	8 (2.0)	390 (97.3)	1	0 (0.0)	0 (0.0)	0 (0.0)	1 (100)
CHADS_2_ or CHA_2_DS_2_-VASc score	401	341 (85.0)	39 (9.7)	10 (2.5)	11 (2.7)	273	255 (93.4)	13 (4.8)	3 (1.1)	2 (0.7)
HAS-BLED score	401	252 (62.8)	87 (21.7)	34 (8.5)	28 (7.0)	207	168 (81.2)	29 (14.0)	10 (4.8)	0 (0.0)
Wells scores for deep vein thrombosis	401	281 (70.1)	58 (14.5)	32 (8.0)	30 (7.5)	184	166 (90.2)	14 (7.6)	1 (0.5)	3 (1.6)
Wells scores for pulmonary embolism	401	193 (48.1)	91 (22.7)	45 (11.2)	72 (18.0)	153	112 (73.2)	29 (19.0)	9 (5.9)	3 (2.0)
Geneva or revised Geneva score	401	1 (0.2)	3 (0.7)	16 (4.0)	381 (95.0)	1	0 (0.0)	0 (0.0)	1 (100)	0 (0.0)
PESI or simplified PESI	401	2 (0.5)	8 (2.0)	16 (4.0)	375 (93.5)	3	1 (33.3)	1 (33.3)	1 (33.3)	0 (0.0)

ACC/AHA = American College of Cardiology/American Heart Association. CPR = clinical prediction rule. JBS = Joint British Societies. PESI = Pulmonary Embolism Severity Index. WHO/ISH = World Health Organization/International Society of Hypertension.

**Table 5.** GPs reporting respective reasons for using clinical prediction rules, *N* = 401

	2014 survey, *n* (%)	2019 survey, *n* (%)
Cardiovascular disease risk	Stroke and venous thromboembolism	Cardiovascular disease risk	Stroke and venous thromboembolism
**Use clinical prediction rule**	395 (98.5)	328 (81.8)	399 (99.5)	398 (99.3)
To aid diagnosis	143 (35.7)	219 (54.6)	145 (36.2)	273 (68.1)
To assess disease severity	191 (47.6)	142 (35.4)	163 (40.6)	141 (35.2)
To guide therapy	336 (83.8)	188 (46.9)	363 (90.5)	278 (69.3)
To guide referral	89 (22.2)	218 (54.4)	95 (23.7)	284 (70.8)
To comply with clinical guidelines or Quality and Outcomes Framework (QOF)	267 (66.6)	106 (26.4)	253 (63.1)	220 (54.9)
To inform or educate patients	220 (54.9)	75 (18.7)	220 (54.9)	137 (34.2)
Automatically generated by practice software	106 (26.4)	19 (4.7)	67 (16.7)	52 (13.0)
Other	1 (0.2)	3 (0.7)	2 (0.5)	3 (0.7)
**Do not use clinical prediction rule**	6 (1.5)	73 (18.2)	2 (0.5)	3 (0.7)

CPRs in other clinical areas GPs most commonly reported using were the Fracture Risk Assessment Tool (FRAX)^53^ (*n* = 24), Centor score^54^ (*n* = 16), FeverPAIN score^55^ (*n* = 16), Patient Health Questionnaire-9^56^ (*n* = 10), Epworth Sleepiness Scale^57^ (*n* = 8), CRB-65 or CURB-65^58^ (*n* = 7), Six Item Cognitive Impairment Test^59^ (*n* = 6), Hospital Anxiety and Depression Scale^60^ (*n* = 6), International Prostatism Symptom Score^61^ (*n* = 6), and QCANCER Risk Assessment Tools^62^ (*n* = 6) (data not shown).

## Discussion

### Summary

The present study evaluated which cardiovascular CPRs are currently recognised and used by GPs in the UK. It also assessed how GPs’ familiarity with and use of cardiovascular CPRs changed by comparing the results of the current and 2014 survey by Plüddemann *et al*.^6^

It was found that cardiovascular CPRs recognised and used by the majority of GPs were QRISK scores, ABCD scores, CHADS scores, HAS-BLED score, Wells scores for deep vein thrombosis, and Wells scores for pulmonary embolism. These cardiovascular CPRs were also the CPRs recommended by UK guidelines and most frequently integrated into GPs’ EHR software. QRISK scores have become dominant CPRs for cardiovascular disease risk assessment in the UK while the popularity of Framingham risk scores waned. For stroke and venous thromboembolism, substantially more GPs are now using ABCD scores, CHADS scores, Wells scores for deep vein thrombosis, and Wells scores for pulmonary embolism. Therefore, it may be hypothesised that integrating CPRs into national guidelines and their EHR software increase the familiarity with and use of CPRs in practice. GPs used CPRs for cardiovascular disease risk most commonly to guide therapy, comply with clinical guidelines, and inform or educate patients; and CPRs for stroke and venous thromboembolism mainly to guide referral, guide therapy, and aid diagnosis.

### Strengths and limitations

To the authors' knowledge, this is the first study that evaluated changes in the familiarity with and use of CPRs over time in a country. Temporal trends were able to be evaluated by applying the recruitment strategy, sampling method, questionnaire, and analysis equivalent to the ones used in the 2014 survey.

Although the study is potentially subject to increased risk of type I error (spurious significant finding) owing to multiple statistical tests, it was found that almost all the comparisons remained statistically significant after adjusting for multiple testing. Only an apparent increased familiarity with the California score did not remain statistically significant and this did not affect the conclusions.

GPs were recruited from doctors.net.uk for compatibility with the 2014 survey. Although this strategy allowed recruitment of a geographically representative sample of GPs from all UK regions efficiently and gave a direct comparability to the 2014 survey, it prevented the response rate from being calculated. The other drawback that could not be excluded was the possibility that the sample over-represented those interested in the topic. It is also unclear whether findings of the survey are generalisable to all GPs in the UK. Furthermore, the findings may have limited applicability outside of the UK and to CPRs for other clinical areas.

### Comparison with existing literature

It was found that most GPs in the survey were familiar with and used cardiovascular CPRs. This is consistent with findings from other recently conducted surveys that reported the awareness and use of cardiovascular CPRs were high.^19,20,26^ For example, a study published in 2015 found 92.5% of Irish GPs were aware of a cardiovascular disease risk calculator and 72.8% of them used it.^19^ On the other hand, studies conducted before 2010 often showed cardiovascular CPRs were infrequently used.^10,12,14,21,22,31^ For example, a US national survey from 2006 found only 17% of family physicians used a coronary heart disease risk calculator.^22^

Common reasons for using cardiovascular CPRs in the existing literature were to educate patients,^17,19,27^ motivate lifestyle changes,^16,19,27^ guide drug therapy,^16,19,23,27^ and establish treatment goal.^19,27^ In addition to these, GPs in the present study frequently used cardiovascular CPRs to comply with guidelines or the Quality and Outcomes Framework, which might be unique for GPs in the UK. The National Institute for Health and Care Excellence (NICE) guideline published in 2008 advised using Framingham risk score to assess cardiovascular risk.^63^ After external validation studies in the UK showed QRISK scores consistently performed better than Framingham risk score,^64–66^ QRISK2 was recommended by the 2014 NICE guideline.^36^ This shift in guideline recommendation might explain notable changes in the use of CPRs for assessing the risk of cardiovascular disease. Similarly, most GPs in the present survey used CHADS scores and HAS-BLED score that were first recommended by the 2012 Scottish Intercollegiate Guidelines Network^39^ and 2014 NICE guideline.^38^

### Implications for research and practice

The study by Plüddemann *et al* ^6^ concluded GPs’ lack of familiarity was one of the reasons for not using CPRs in practice. Findings of the present study suggest that integrating CPRs into national guidelines and EHR software might be important factors for increasing GPs’ familiarity with and use of CPRs. Conducting an international survey in countries where guidelines recommend different CPRs and where EHR software has a varying degree of CPR integrations might be useful in assessing these associations. Ultimately, the hypotheses could be tested by interrupted time-series studies and comparisons between countries with different guidelines or institutions with different CPRs integrated in EHR software.
