# Correction: GPs’ familiarity with and use of cardiovascular clinical prediction rules: a UK survey study

**DOI:** 10.3399/bjgpopen21X101162

**Published:** 2021-04-23

In the Research article by Ban *et al.* GPs’ familiarity with and use of cardiovascular clinical prediction rules: a UK survey study *BJGP Open* 2020; DOI: https://doi.org/10.3399/bjgpopen20X101081, the Funding section was incomplete, stating: “This study was funded by the National Institute for Health Research (NIHR) Collaboration for Leadership in Applied Health Research and Care Oxford.” This section should have stated: “This study was funded by the National Institute for Health Research (NIHR) Collaboration for Leadership in Applied Health Research and Care Oxford. RP acknowledges part-funding from the NIHR Oxford Biomedical Research Centre, the NIHR Oxford and Thames Valley Applied Research Collaborative (ARC), NIHR Oxford Medtech and In-Vitro Diagnostics Co-operative and the Oxford Martin School and part-funding from the National Institute for Health Research (NIHR Programme Grant for Applied Research)."

We apologise for this error. The online version has been corrected.

